# Is asthma mortality decreasing or increasing in Brazil? The burden of proof

**DOI:** 10.36416/1806-3756/e20240355

**Published:** 2024-11-16

**Authors:** Marcia M M Pizzichini, Álvaro A Cruz, Emilio Pizzichini

**Affiliations:** 1. Programa de Pós-Graduação em Ciências Médicas, Universidade Federal de Santa Catarina, Florianópolis (SC) Brasil.; 2. Programa de Pós-Graduação em Medicina e Saúde, Faculdade de Medicina da Bahia, Universidade Federal da Bahia, Salvador (BA) Brasil.; 3. Programa para o Controle da Asma na Bahia - ProAR - Universidade Federal da Bahia, Salvador (BA) Brasil.

Asthma affects over 350 million people globally and is the most common chronic respiratory disease in children and young adults, although it affects people of all ages and it is an important cause of premature death. Also, it poses a significant public health challenge across and within different countries, with prevalence rates varying widely, but with a trend toward a decrease in its prevalence and mortality in several countries.^1^ Therefore, it is important to monitor both the similarities and differences in asthma indicators across Brazil, a country characterized by significant social inequalities on top of regional economic and educational disparities.

Although asthma-related deaths are rare, they are unacceptable, as the disease is easily treatable for most patients and these fatalities are largely preventable. Most asthma-related deaths occur in low- and middle-income countries, or low-resource settings, highlighting a critical need to enhance our understanding of the risk factors, including issues about access to proper health care and management of various asthma phenotypes and endotypes.^1^ Furthermore, improving the assessment of asthma’s causes and treatments in less economically developed regions of the globe could lead to a deeper understanding of how equity in treatment access impacts on asthma control and remission.

A major disproportion between asthma prevalence, mortality, and morbidity still exists in many countries and must be addressed. For example, according to the publication “Global Burden of 369 Diseases and Injuries in 204 Countries and Territories, 1990-2019: A Systematic Analysis,” released in 2020,^2^ asthma ranked 24th among noncommunicable diseases in terms of disability-adjusted life years (DALY) for individuals aged 0-9 in 1990, moving to 19th in 2019. For the age groups 10-24, 50-74, and ≥ 75 years, there were notable changes: rankings shifted from 23rd to 27th, 15th to 28th, and 18th to 24th, respectively. Despite the noticeable improvement in asthma-related DALYs in the ages over 9 years, the burden of the disease remains significant.

Recently, two significant observational studies analysing trends in asthma mortality in Brazil were published in the JBP,^3^ ^,^ ^4^ both authored by experts in the field. Yet they arrived at different conclusions. Pinheiro et al.^3^ employed secondary data from the Information Technology Department of the Brazilian Unified Health Care System to estimate proportional hospitalization and in-hospital death rates per 100,000 population. Their analysis covered the years 2008 to 2021 and revealed that Brazil experienced over 8,000 deaths and more than 1.7 million hospitalizations due to asthma during this period. Particularly, both the number of deaths and hospitalizations significantly declined over the study years. On average, this corresponded to approximately one death per day and over 55,000 hospitalizations annually, with a mean length of hospital stay of three days.

Brum et al.^4^, in a separate retrospective observational study, sourced their data on asthma mortality from the *Sistema de Informações sobre Mortalidade do Ministério da Saúde do Brasil* (SIM, Brazilian National Ministry of Health Mortality Database) covering the period from 2014 to 2021. This database is accessible at https://opendatasus.saude.gov.br/. The study population comprised all asthma-related deaths (ICD-10 codes J45 and J46) among individuals over 6 years of age.

During the study period, Brum et al.^4^ reported a total of 18,854 asthma-related deaths in Brazil, with an annual increase of 2.6%, equating to 0.04 additional deaths per 100,000 population (95% CI, 0.02-0.06; p < 0.01). The northeastern region exhibited the highest prevalence of asthma deaths, at 1.60 deaths per 100,000 population, while the southern region experienced the greatest increase over the study period (37%). A higher proportion of deaths occurred among females and elderly patients. Notably, when analysing the location of these asthma-related deaths, it was found that 28% occurred at home. The authors also indicated that asthma death rates increased progressively over the years, with the highest peak occurring in 2020.

At first glance, the apparent contradictions between the two studies may be attributed to their data sources of secondary data: Pinheiro et al.^3^ examined intra-hospital asthma deaths, while Brum et al.^4^ analysed all death certificates in which asthma was the primary cause of death. Both forms of data registration may have inaccuracies that could not be prevented and impact the results of the studies. However, one could argue that these studies are not directly comparable due to the differing sources of data utilized in each.

Likewise, data source may not be the only explanation, as the significant disparity in reported asthma death rates-approximately one death per day in the first study^3^ compared with six deaths per day in the second-suggests that additional contributing factors might be involved. Besides, it is important to emphasize that, in both studies, the authors thoroughly discussed their findings, along with the strengths and limitations that could have influenced the results.

One notable aspect of the study by Pinheiro et al.^2^ is the significant decline in hospital admissions-73% over the years-excluding the COVID-19 pandemic years. This decline has occurred progressively since the introduction of asthma medication at no cost in the Brazilian public health care system in 2008.^5^ Consequently, the extent of this reduction in hospital admissions suggests that the corresponding decrease in hospital deaths due to asthma may primarily be associated with improved asthma management and a reduction in hospitalizations.

On the other hand, the study by Brum et al.^4^ indicated that asthma death rates increased progressively over the years, reaching their highest peak in 2020, coinciding with the onset of the COVID-19 pandemic. The authors note that, despite excluding deaths directly related to COVID-19, the restricted access to health care during the pandemic may have influenced this particular peak.

Another factor that may have influenced the results of the study by Brum et al.^4^ is that approximately one-third of the asthma-related deaths occurred at home, where determining the true primary cause of death may be complicated by potential over- or underdiagnosis of asthma. As the authors noted, it is essential to develop better public health care strategies to prevent such unacceptable outcomes. Additionally, the higher mortality rates among the elderly must be considered, as existing comorbidities can significantly impact asthma control and/or may contribute significantly to the deaths.

In summary, JBP published two distinct observational studies that reached different conclusions. These discrepancies may be attributed to the varying study source of information, and samples and should be confirmed by future research. It is important to highlight that, despite the significant decline in in-hospital asthma death rates, the overall rates of asthma-related deaths might be increasing, underscoring the need for improved public policies, as it suggests poor perception and possible negligence in handling emergencies in asthma at home.^6^
